# Cloud-based relational database for multimodal animal data

**DOI:** 10.1093/database/bay124

**Published:** 2018-12-14

**Authors:** Niklas Pallast, Frederique Wieters, Marieke Nill, Gereon R Fink, Markus Aswendt

**Affiliations:** 1 Department of Neurology, University Hospital Cologne, Cologne, Germany; 2 Cognitive Neuroscience, Institute of Neuroscience and Medicine (INM-3), Research Center Juelich, Juelich, Germany

## Abstract

Pre-clinical research builds on a large variety of *in vivo* and *ex vivo* tools such as non-invasive imaging, microscopy, and analysis of gene expression. To work efficiently with multimodal data and correlate results across scales, it is of particular importance to have easy access to all data points from different specimen, e.g. the magnetic resonance imaging (MRI) data from different time points, and the post-mortem histology. That requires an efficient data management, which is customizable and designed to relate all applied methods, raw data and analyses to one specific animal. Despite increasing demands to handle such complex data, most pre-clinical labs have not yet established such an electronic database. Here, we present a novel cloud-based relational database for multimodal animal data, which operates on commercial software. We have implemented data fields for various pre-clinical features such as MRI, histology and behaviour. Automated procedures replace manual and recurrent calculations. Pre-set plotting and printing features provide efficient analysis and documentation. The database template is useful for all labs working with laboratory animals and the adaption to specific research projects requires no prior scripting expertise. The database works operating-system independent through the web browser and allows multiple users to work simultaneously. The data entry is monitored and restricted for particular tests according to the user management in order to keep for example users during the experiment blinded for the experimental group. The database improves data accessibility, standardization of data recording and data handling efficiency in pre-clinical research.

## Introduction

To obtain valid scientific results in pre-clinical research, standardization of experimental protocols and data handling need to be set before starting the actual experiment. Efficient data management becomes more and more important with the increasing number and variety of experimental procedures. However, to date, preclinical research is still in a transition phase. While the recorded data type is predominantly electronic data, the documentation is still the lab notebook. Electronic data can be shared with many users at any time, however, the lab notebook remains in the lab together with the documentation about the metadata. Thus, the link to the actual research project and subject is often difficult and sometimes even impossible to restore from an old lab notebook. An experiment involving 100 animals in two subgroups, which received six test procedures at five different time points, will already add up to 3000 data points in total. Keeping track of such large multimodal data constitutes a major challenge for all labs and a necessity for the success of all big data science initiatives, e.g. in neuroscience to map the brain at different scales and correlate gene expression and electrophysiological measurements (1).

Good scientific practice requires easy access and safe data recording and storing. However, many researchers underestimate the difficulty of retaining the relationship between individual data points. For example, data management, if pursued with the traditional handwritten method becomes prone to user errors and should be replaced by an electronic system, which also monitors access to the data and changes thereof. Electronic databases have been developed predominantly for collecting data or providing a platform for uniform analysis (Table 1). The Open Microscopy Environment Remote Objects (Omero)—for microscopy (2), the Picture Archiving and Communication System (PACS)—for radiological data (3) and the International Mouse Phenotyping Consortium (IMPC)—for mouse strains and phenotypes (4), are examples of efficient tools to facilitate data sharing for researchers across disciplines. However, for day-to-day experimental data management, recording and analysis, there is only a very limited number of options available. The Research Electronic Data Capture (REDCap) project (5) provides a web-based software for collaborative research studies and supports data collection, storing and sharing using PHP/Java and MySQL scripting language. Furthermore, there are commercial solutions such as electronic lab notebooks (eLNs) (6), which are optimized for the workflow of `wet-labs’, performing cell and molecular biology experiments (Table 1). Notably, most tools do not support the relational database model for animal research, which was introduced by E. F. Codd in 1970 (7). Here, the investigated facts are linked without contradiction and permanently to the corresponding data, which is crucial for retrieving information efficiently through a search function. Through that operation, it is possible to identify the subject that received a specific treatment at a particular time point and correlate the measurement results to the related subject immediately. Furthermore, there is no database solution available that supports the simultaneous operation by many users and the association of large amounts of multimodal data with individual animals and different experimental groups (e.g. treatment vs. placebo). This setting is not exclusive to our lab, it is similar for all labs working with animals and is only different for the tests being applied.

**Table 1:** Comparison of tools for research data management and analysis

**Software**	**Description & features**	**Costs**	**Access**	**Security**	**Infrastructure**
**Ninox**	Pre-clinical data management, electronic data capture, analysis and reporting	7€ per user/ month	Web, iOS App	SSL/TLS – 2048bit	Various server locations in Europe
**OpenClinica**	Clinical data management and electronic data capture, randomization, supply management	n.a.	Web	SSL – n.a.	n.a.
**Omero**	Open microscopy environment for viewing, organization, analysis and sharing of microscopy data	0€	Web, Windows/Mac/Linux clients	SSL – n.a.	Self-hosted institutional server
**REDCap**	Design and management of online surveys and databases	0€	Web	SSL – n.a.	Self-hosted web/database/email server
**PyRAT**	Python-based relational animal tracking for animal facility management	n.a.	Web	SSL – n.a.	Self-hosted Linux or Unix based server with support for Python and MySQL
**SciNote**	Electronic lab notebook, inventory management, user management	0€ for 1 team	Web	SSL 256bit	Heroku PostgreSQL database and Amazon S3
**Benchling**	Electronic lab notebook, note-taking, sample tracking (focus on molecular biology)	0€ for academics	Web	SSL 256bit	Amazon S3
**labfolder**	Electronic lab notebook, import for various file formats including images and Word/Excel, user management	15€ per user/month (group of max. 3 is 0€)	Web, iOS + Android App	SSL 256bit	Server location n.a. or Self-hosted

We use a multitude of biological imaging and analysis methods and apply them to study structural and functional recovery after experimental stroke in mice. In this context, we face the following major challenges in our daily routine: (i) At which timepoint was the data acquired? (ii) Who acquired the data? (iii) Where is the data stored? (iv) Who did the evaluation and analysis? (v) To which experimental group does a given mouse belong? Lab notebooks and the currently available electronic tools are not applicable to such a complex workflow. The database presented here provides a user-friendly and highly flexible environment for animal research.

## Database design and implementation

We designed a cloud-based relational database with tools provided by commercial software (Ninox Software GmbH, Berlin, Germany) with the aim to record a multitude of different experimental procedures. The database software is available as cloud and self-hosted server solution with SSL-secured web interface and iOS App (Table 1). It features data management and data storage, user rights management, local backups and history tracking. All files can be saved in the database directly; however, for large files we recommend to store them on a central file server and provide the file link in the database. Using the Ninox software we have established (i) a comprehensive pre-clinical data and project management tool for animal experiments including calculation of animal numbers and generation of unique identifiers for experimental groups, (ii) a database structure which is adaptable and changeable by an interactive user interface without any scripting knowledge, (iii) a standardized electronic data capture with interactive fields, automated calculations, time stamping and data lock, (iv) a user rights management for entry-selective read/write permissions (necessary to make the experimenter `blinded’, e.g. for the treatment vs. placebo group) and (v) a search, print, chart and report function.

### Project planning

**Figure 1:**
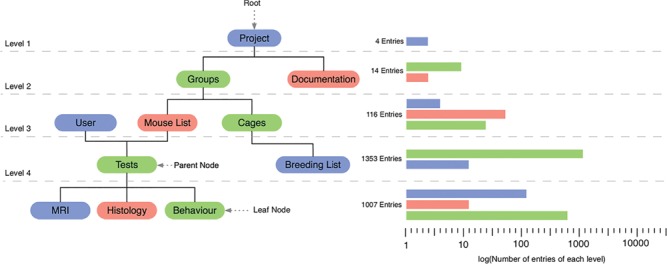
Graphical illustration of the database relations shown as tree structure. The root of the tree is represented by the entity `project’ and the leaves represent different test procedures. The bar graph represents an example of the number of entries for each level.

In our case, we collect the following *in vivo* and *ex vivo* biomedical data from mice: type of surgery, behavioural tests and scoring (cylinder test, rotating beam test, grid walk test, corner test), magnetic resonance imaging (MRI) and histology (stainings and microscopy). The different input data and their associated procedures were set in relation to each other and transferred to the Ninox software as illustrated by the tree structure in Figure 1. The root of the structure is the general project to which different subgroups (sub-projects) belong, for example sham and non-sham groups. Each group has an associated number of rodents listed in the mouse list. Each mouse is identified by an experiment-specific ID, which relates to the project, the subproject, the mouse cage and the mouse number and is being automatically concatenated, e.g. the project drug test (DT) with the subproject aspirin type 1 (A1) and mouse 1 from cage 5 is shortened to DT_A1_5_1. Just as each group owns several cages, one mouse receives several tests from different users. Each test may be composed of different procedures, e.g. behavioural testing, MRI and histology, which are represented by leave nodes (Figure 1). The data of these tests are inherently linked to the parental entities of the mouse over four levels. The tree structure reduces the error rate during data collection since duplicate or missing data are easily identified. Through the automatically generated field `Related Event + Mouse’ the user keeps control that the correct test is related with a specific mouse. Only the admin is allowed to correct that relationship if necessary.

**Table 2:** Overview of the information fields of a single mouse in the mouse list. Each field has an associated data type: date (d), choice (c), number (n), formula (f), string (s) and multiple choice (mc)

**Name**	**Datatype**	**Explanation**
***Mouse data***		
Birthdate	date MM/DD/YYYY	The day the animal was born
Sex (c)	male/female	Allocate sex
Mouse registration fate (d)	date MM/DD/YYYY	The day the animal was included in study
Initial weight (n)	Floating point in gram	Weight of the animal at registration date
Cage number (n)	Integer	Related cage number
Tail lines (s)	Number of lines	Helps to identify the animal in the cage
Distributor (mc)	String	Animal distributor
***Mouse status***		
Lifetime in weeks (f)	Weeks	Time elapsed between birth and death
Dead or alive (c)	Dead/alive	To allocate date of death
Cause of death (mc)	Perfusion/died in Experiment	To differentiate perfused mice from unintended deaths, e.g. during an experiment
***Study Information***		
Access locked (c)	Locked/open	When `locked’ no user than the administrator can change the data
Date of locked access (d)	date MM/DD/YYYY	Date the access has been changed
Included in study (c)	Yes/no	Administrator/project leader determines if animal is included or excluded from the study
Study comment (c)	String	Study-related comments, e.g. why an animal was excluded
***Administrative information***		
Group (s)	String	Related group
Study ID (s)	String	Experiment-specific ID
Registered by (mc)	Name of the user	User, who registered the animal in the database
Team leader (s)	String	Responsible person
Animal permission (s)	Integer	Administrative number
Workgroup (s)	String	Related workgroup

### Data entry

As the database is available online and synced automatically, the user can enter new data into the uniform structure via pre-defined information fields already during an experiment. The mandatory fields for a specific test are highlighted in red. Which tests are available is being defined by the administrator. For example, the *ex vivo* test `Histology’ becomes only available when the time point `ex vivo’ is selected. As an example, the fields of the entity `Mouse List’ are listed in Table 2. These fields can be changed and easily adapted to other experimental workflows and methods. The information is stored for every single mouse and is related to different tests at particular days. All data, such as `Birthday’ of the mouse and `Registration date’, are simple information fields, while, for example `Lifetime’ is a formula field, which calculates the current age of the animal based on the date and the date of birth automatically. Calculation fields and smart buttons were implemented to provide standardized electronic data capture with interactive fields and the evaluation of data directly in the database (Table 3).

**Table 3:** List of representative calculations for different entities, which were automated to replace repetitive and manual calculations

**Entity**	**Input data**	**Output data**
Grid walk test	Foot fault, # of total steps	Percentage of foot faults
Corner test	Right turn, left turn	Percentage of turn distributions
Scoring	Weight, general and focal deficits	Overall deficit level, weight change (%)
Rotating beam test	Distance, time, hindlimb displacement, animal drops	Avg. speed, avg. distance, avg. hindlimb drops
Mouse list	Birthdate, date of death	Lifetime
Projects	Number of animals in project	Animal permission-related number of remaining mice in project

**Figure 2:**
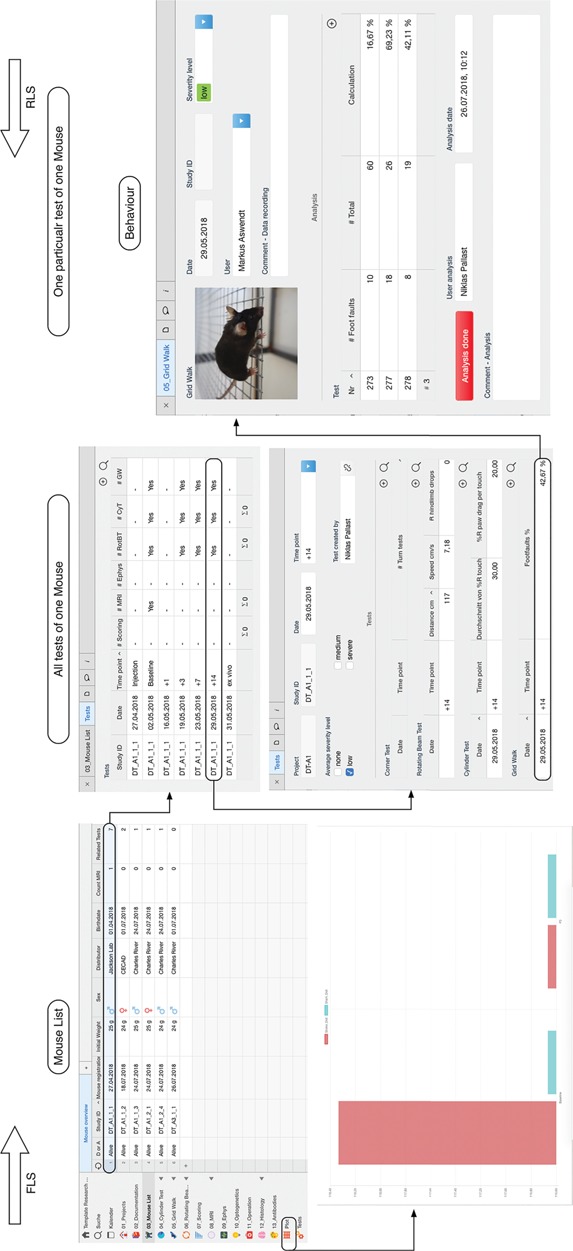
In the database model, a single mouse in the `Mouse List’ is related to different tests. These tests represent the *in vivo* and *ex vivo* experiments and are summarized in the `Tests’ table view. By selecting a specific data, it is possible to navigate from the mouse list to the specific test, in this example the grid walk test and access the raw data. The data entry is confirmed and signed electronically with the button `Analysis done’ (right). The plot function is available for a single mouse experiment or through the specific plot feature for a group of selected mice (bottom left). In this example, the average value for the distance walked over the rotating beam is plotted for a stroke and sham surgery group of mice.

**Figure 3:**
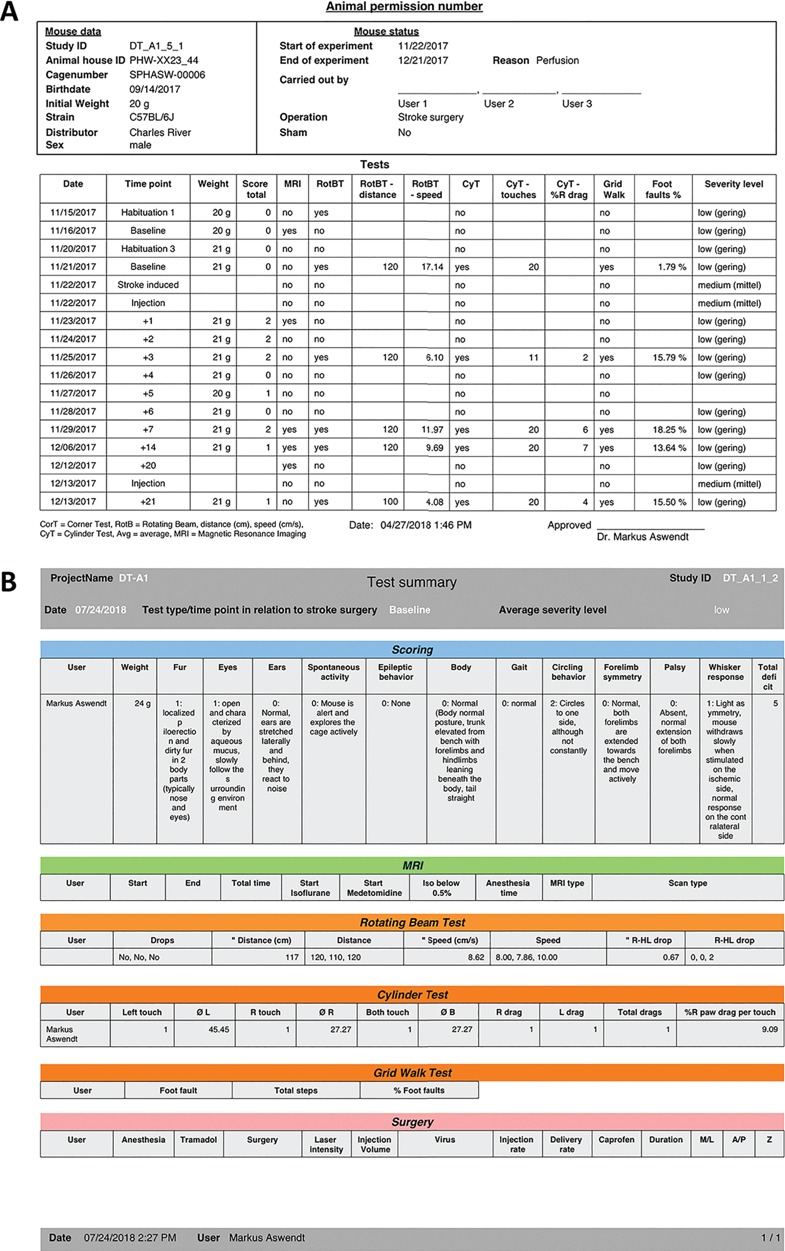
Report function for animal data contains either an overview with all tests performed with one subject (A) or all details for a selected time point including the raw data and analysis (B).

### User management

The user rights management is based on four hierarchical levels: the owner, the administrator, the editor and the guest. It is necessary that only one user, the owner, is allowed to invite new users and has full control over all features of the database. The administrator is allowed to assign read/write permissions, delete data and modify information fields. To ensure that data has not been altered by an unauthorized person, the rights management allows all editors only to edit certain entities as long as the experiment is not completed. At the lowest level guest are associated, who are not allowed to change any data, but they have read permission. We have implemented two safety procedures to avoid manipulation and false entries. Firstly, when the data entry is finished, the editor confirms the process with the button `Analysis done’. The trigger will automatically sign the data entry with user name and time stamp and lock the data entry field (Figure 2). Thus, the data entry remains visible in the table view, however, only accessible for the administrator. Secondly, for all tests that require the data fields to be entered during the experiment (for example MRI), the fields are accessible for the editor only once. Similar to the button `Analysis done’, a question button `Done?’ appears in the MRI field after the last field is entered by the user. By confirmation, the data acquisition is automatically signed electronically and only the admin is allowed to modify these fields later on. Thirdly, if an animal study is completed, only the administrator is allowed to close the data entry with a lock command (table view `Mouse List’) and sets it to read-only for all editors. As long as this lock is not set, the editor is kept blind to the experimental condition (e.g. treatment vs. placebo).

### Search and filter function

Another advantage of the database structure is the bidirectional search for related data. The Reverse Level Search usually starts intuitively with an entity with fewer information fields. It starts at a leave node and runs step by step to higher level orders (Figure 2). If the *a priori* properties are known about a study, the Forward Level Search is applicable. That improves the project management, as the administrator can quickly check on the current experimental progress, e.g. on how many animals of one group have been used and what was the outcome in order to draw conclusions without having to wait for other users to export, prepare and send the data. It allows the users to search for mice of a specific group (e.g. all in project V1), which received a particular test in order to export data for further analysis. This way it is also possible for the user to identify missing entries, e.g. by applying the table view filter for a specific subject and list all time points of a specific test.

### Plot function

The database provides a pre-assembled plot function to visualize data from selected behaviour tests and average values across subjects (Figure 2). Each user can select specific mice for the plotting. However, we have implemented the average function in a way, that user with the role `editor’ remain blinded for the experimental group (e.g. stroke or sham surgery). Thus, in the final graph, only the experimental groups are listed, not the individual study IDs. The Ninox software provides pre-set functions to change the graph design (bar, line graph and others).

### Report function

To document the electronic animal records in case of a data loss and for animal permission-sensitive data, we adopted print functions to summarize relevant information in one PDF file (Figure 3). The animal permission-sensitive number of approved and used animals is automatically calculated based on the initial experimental planning. It is possible to export the information of single tests and all tests that have been done with one subject. In addition, there is an export function for selected data (as text, CSV and Excel document). There is the possibility to manually back up the database from the cloud to the local machine.

## Discussion

Although outlined in the Good Laboratory Practice (GLP) guidelines of the WHO (8), to date most labs store their data not in a standardized way and lack way behind clinical standards such as Good Clinical Practice compliant data management (9). This represents a major drawback for attempts to improve standardization, reuse of data and data reliability. The current standard, the laboratory notebook, is not appropriate for data science-driven approaches and prevents (meta) data search and reuse. Most importantly, a written documentation or the most widely used Excel table does not provide information regarding the relationship between different experiments at different days and the associated animal(s). The specific experimental workflow for animal experiments is not fully integrated in existing databases and eLNs. The current software either supports only data storage and retrieval (PACS for radiological data), or provides a complete package of data organization, analysis and sharing, however, only for a single data type (Omero for microscopy). Furthermore, data sharing tools with a focus on animal data only comprise online resources and exchange platforms for example IMPC, the mouse web portal (4) of transgenic mice and related phenotyping data or animal facility management tools such as the Python-based relational animal tracking software (PYRAT). The relational database presented here was specifically designed to fill an existing gap and improve reliability and validity of animal research. It provides a cloud or self-hosted server project and data management system for multimodal and longitudinal animal experiments. The design of the database is in agreement with the guidelines of GLP (8) and Findable, Accessible, Interoperable, Reusable (FAIR) guidelines (10): (i) there is a unique identifier for each mouse, (ii) the (meta)data for each test is (manually) recorded or linked and (iii) all data entries include qualified references to related (meta)data, e.g. the experimental group. The database design is applicable to all research groups working with laboratory animals. It is cost-effective and can be easily adapted to other test procedures and requires no prior knowledge in database design or programming skills in database languages such as My Structured Query Language (MySQL). The connection to the `cloud’ server hosted by Ninox and located in different locations in Europe is SSL (2048-bit) encrypted. The server is compliant to the strict General Data Protection Regulation by the European Union. It is possible to back up the data and export the database as separate tables. Nevertheless, we advise users to store the actual raw data on a dedicated central file server (e.g. a local network-attached storage). That ensures data security and control over the data. If necessary, Ninox such as other database tools, can be installed on a dedicated server, however, in this case the user is responsible for maintenance and database integrity.

One of the major challenges of a pre-clinical database is the variety of *in vivo* and *ex vivo* tools. Currently, the data entry is mainly manually and standardized by a certain structure in order to keep most flexibility for further adaption. Additional features, such as the automated reading of image headers, e.g. from the DICOM format, for a more advanced analysis need to be implemented by the user. We are using the database with a team of 6 people and manage 4 different projects, 10 experimental groups and more than 100 mice acquired during the last 10 months. On average, 14 tests were conducted with each mouse, giving a total of 1033 tests. In contrast to the conventional written documentation or data management using Excel, the database improved data accessibility, reporting, efficiency to search and collaborate, and reliability of experimentation. Furthermore, the results were immediately available for the project-responsible person. The database template is available as supplement. Continuous updates will be available from our website and the official Ninox website, and we will help other researchers to adapt the database to their needs.

## Supplementary Material

Supplementary Data

## Notes

**Database URL:** https://neurologie.uk-koeln.de/forschung/ag-neuroimaging-und-neuroengineering/
